# The hidden risk factors behind of suicidal behavior in medical students: a cross-sectional cohort study in Mexico

**DOI:** 10.3389/fpsyt.2025.1505088

**Published:** 2025-03-05

**Authors:** Margarita L. Martinez-Fierro, Jorge R. Reyes-Hurtado, Anayantzin E. Ayala-Haro, Lorena Avila-Carrasco, Leticia A. Ramirez-Hernandez, Georgina Lozano-Razo, Javier Zavala-Rayas, Sodel Vazquez-Reyes, Alejandro Mauricio-Gonzalez, Perla Velasco-Elizondo, Vladimir Juarez-Alcala, Ricardo Flores-Vazquez, Ivan Delgado-Enciso, Iram P. Rodriguez-Sanchez, Idalia Garza-Veloz

**Affiliations:** ^1^ Doctorado en Ciencias con Orientacion en Medicina Molecular, Academic Unit of Human Medicine and Health Sciences, Universidad Autonoma de Zacatecas, Zacatecas, Mexico; ^2^ Unidad Academica de Matematicas, Universidad Autonoma de Zacatecas, Zacatecas, Mexico; ^3^ Unidad Academica de Psicologia, Universidad Autonoma de Zacatecas, Zacatecas, Mexico; ^4^ School of Medicine, University of Colima, Las Víboras, Mexico; ^5^ Laboratory of Molecular and Structural Physiology, Universidad Autonoma de Nuevo Leon, San Nicolas de los Garza, Mexico

**Keywords:** suicide, suicidal behavior, anxiety, depression, medical students, addiction, tobacco use, alcohol abuse

## Abstract

**Introduction:**

Suicidal behavior among medical students is a significant concern, requiring a thorough understanding of effective intervention and prevention strategies. This study aimed to generate a situational diagnosis and establish the risk factors associated with suicidal behavior among medical students.

**Methods:**

In a cross-sectional cohort design, we surveyed 688 medical students in Zacatecas, Mexico, employing 14 validated questionnaires to assess suicidal behavior, aspects of their lifestyle, perceived support, risk factors including mental health disorders, and substance use. Univariate and multivariate analyses were performed to examine the associations between the study variables and suicidal behavior.

**Results:**

Suicidal behavior was associated with the following variables: female sex, non-heterosexual orientation, history of psychiatric illness, childhood trauma, bullying, symptoms suggestive of attention-deficit/hyperactivity disorder (ADHD), and contexts of substance use such as unpleasant emotions (p <0.05). Multivariate analysis revealed that mild tobacco use, alcohol consumption, severe hopelessness, family history of mental disease, material, and affective support significantly increased the odds of suicidal behavior (OR values: 1.56–8.78, p <0.05). Anhedonia, sexual orientation, and problematic consumption of cannabis were significantly associated with suicide attempts, with higher OR of 9.92, 6.49, and 5.56, respectively.

**Conclusions:**

Sexual orientation, substance use, lack of material, and affective support were identified as significant risk factors for suicidal behavior and suicide attempts among medical students. Additionally, hopelessness, history of mental health diseases, and ADHD symptoms were associated with an increased risk. These findings underscore the need for targeted interventions that include behavior modification for substance use and the reinforcement of emotional and social support networks.

## 1 Introduction

Suicidal behavior, a spectrum of actions including ideation, self-harm, attempts, and suicide, represents a growing concern in global public health. The World Health Organization (WHO) reports that suicide is among the leading causes of death worldwide, with approximately 703,000 individuals taking their own lives annually (1). It is estimated that 30 more individuals attempt suicide, highlighting the magnitude of this issue (2). Worldwide, among the university-aged population, suicide is the second leading cause of death, with a mortality rate of 7.5 per 100,000 university students (3).

Suicidal behavior results from a complex interaction of psychological, social, biological, cultural, and environmental factors (4). Known risk factors associated with this behavior are diverse, ranging from mental health disorders such as depression, anxiety, bipolar disorder, and attention-deficit/hyperactivity disorder (ADHD), adverse socioeconomic circumstances, traumatic experiences, to access to lethal means (5, 6). From a pathophysiological standpoint, suicidal behavior is linked to alterations in brain neurotransmitters, such as serotonin, which affect mood, sleep, and appetite regulation. These neurochemical changes, combined with genetic and environmental factors, contribute to individual vulnerability to suicide (4, 7). In young adults, these factors can be intensified by the unique pressures of this life stage, including academic stress, interpersonal relationships, and career expectations (8).

Numerous risk factors for suicidal behaviors have been identified among medical students. For instance, a large-scale study conducted in Brazil with 4,840 medical students sought to identify factors associated with a history of suicide attempts and found an 8.94% prevalence of suicide attempts among this group (8). Their results identified female gender, homosexuality, low income, bullying by university peers, childhood or adult trauma, family history of suicide, recent suicidal ideation, daily tobacco use, and severe risk for alcohol abuse as significant predictors (all p <0.05), with an area under the curve of 0.83 (8). Another meta-analysis among African medical students showed that female sex, alcohol use, depression, and khat use were significantly associated with suicidal ideation, while depression was also associated with suicide attempts (9). While risk factors such as depression and loneliness are well-established contributors to suicidal behavior among university students, protective factors such as social support play an equally important role in mitigating these risks. Higher levels of perceived social support have been associated with lower rates of depression and suicidal ideation in this population (10, 11), emphasizing the importance of promoting supportive networks as a strategy to reduce the prevalence of mental health disorders among university students.

In Mexico, medical students face a high stress burden due to the academic and practical demands of their training (12). It has been observed that these students may be at an increased risk of developing suicidal behaviors due to factors such as emotional exhaustion, limited free time, and restricted access to adequate mental health services (8). A study of first-year Mexican students showed that 27.6% had mental health problems and 2.4% were classified as serious cases. Moreover, depression in medical students was higher than that in the general population; 23% of first-grade students presented symptoms and were associated with poor academic performance (13). The COVID-19 pandemic exacerbated this situation, leading to a significant rise in anxiety and depression disorders worldwide (12, 14, 15). Social isolation, economic uncertainty, and fear of disease have created breeding grounds for mental health deterioration (12, 14). With the aim of assessing suicidal behavior among Mexican medical students Galvan-Molina et al. (16) surveyed 323 participants across three academic levels and found a suicide risk of 9.6%, a high prevalence of attention deficit disorder with hyperactivity (ADHD; 27.9%), depression (23.8%), anxiety (13.3%), and a 13.3% rate of high-level Burnout. They also noted detrimental use of tobacco and alcohol in one-fifth of the sample. In a larger study (n = 8,858), Escobar-Padilla et al. (17) examined three cohorts of undergraduate Mexican medical students as they began their internship years, reporting that 37.2% had severe anxiety, 14.9% had moderate or severe depression, and 8.5% had suicidal ideation. Female sex and enrollment in a private university were linked to an increased risk of anxiety and depression, while high violence zones, severe anxiety, or depression elevated the risk of suicidal ideation (17). Although these studies have provided valuable insights into the mental health challenges faced by Mexican medical students, they also present notable limitations that warrant further exploration. For instance, Galvan-Molina et al. focused largely on the prevalence rates of psychopathology without examining the underlying risk factors for suicidal behavior, whereas Escobar-Padilla et al. employed a narrower range of inventories that omitted key contributors, such as ADHD symptoms, hopelessness, anhedonia, or substance use. None of the studies specifically investigated suicide attempts or self-harm behaviors (integral components of suicidal behavior) and excluded contextual factors such as perceived social support, social class, and/or sexual orientation. These gaps highlight the need for more complete research that integrates a wider array of validated instruments, thereby allowing for a deeper understanding of how individual, cultural, and social risk factors intersect to influence the spectrum of suicidal behaviors, including ideation, attempts, and self-harm.

Given the limited research specifically focused on medical students in Mexico, our study aimed to generate a situational diagnosis and identify the risk factors associated with suicidal behavior among medical students. We hypothesized that factors such as female sex, non-heterosexual orientation, history of psychiatric illness, childhood trauma, bullying, presence of mental disorders, tobacco use, alcohol and cannabis consumption, family history of mental disease, anhedonia, and/or insufficient material and social support are significantly associated with increased suicidal behavior in this population. To test these hypotheses, we employed a multidimensional approach, including univariate and multivariate analyses, to identify significant associations between the hypothesized risk factors and suicidal behavior. Data were collected using validated instruments, including drug use inventories, mental health screening tests, and surveys assessing personal history of abuse/trauma, physical activity, and perception of social support. Through this study, we aimed to provide a foundation for effective prevention and intervention strategies tailored to the specific needs of this vulnerable population, addressing a critical gap in the existing literature while offering valuable insights for health professionals, educators, and policymakers.

## 2 Materials and methods

### 2.1 Study design and participants

This study employed a cross-sectional cohort design utilizing surveys to collect data from participants at a single point in time. It was conducted in Zacatecas, Mexico, from August to December 2023. The study population consisted of students from the General Medicine Program of the Academic Unit of Human Medicine and Health Sciences of the Autonomous University of Zacatecas “Francisco García Salinas” (18). This study was guided by a biopsychosocial model that considers biological, psychological, and social factors to understand suicidal behavior. Participants included in the study met the following selection criteria: students of the General Medicine program who were enrolled in their academic plan at the Academic Unit of Human Medicine and Health Sciences, regardless of sex, aged between 18 and 35 years, and who agreed to participate in the study by signing an informed consent form. The participants did not receive any compensation for their participation. Participants who did not complete the inventories, withdrew from the study, or for whom more than 80% of the required information was not available were excluded from the study.

### 2.2 Sample and sampling

Stratified random sampling with proportional allocation was then performed. The strata considered were related to contextual factors associated with suicidal behavior: sex, foreign status, campus, and semester. A population size estimated of 2,636 students was considered, with a minimum probability of 6.9% for suicidal ideation (19), 15.4%–19.5% for depression, 19.3%–31.3% for anxiety (20), and 8.8% for ADHD (21). A sample size of n = 621 was estimated, with an expected maximum error of 5%. For sampling, data projections on program enrollment were utilized, and the calculation of overall and stratum sample sizes was performed using Excel ^®^ software (Microsoft Office).

### 2.3 Techniques and instruments for data collection

Data were collected through a web-based survey using questionnaires to assess the different risk factors. Fourteen instruments were used, including the Beck Scale for Suicidal Ideation (22), Beck Hopelessness Scale (23), Depression, Anxiety, Stress Scale (DASS-21) (24), Plutchik’s Impulsivity Scale (25), International Physical Activity Questionnaire (IPAQ) (26), Adult ADHD Self-Report Scale (ASRS V.1.1) (27), Social Support Survey (MOS) (28), Alcohol Use Disorders Identification Test (AUDIT) (29), Self-Injury Questionnaire (SIQ) (30), Cannabis Addiction Test (CAST) (31), Fagerström Test for Nicotine Dependence (FTND) (32), Cocaine Addiction Test (ASSIST) (33), Drug Use Situations Inventory (DUSI) (34), and Reasons for Attempting Suicide Questionnaire (RASQ) (35). Each instrument was validated in previous studies (36–50).

### 2.4 Procedures for instrument administration

The measurement instruments were administered anonymously to protect participants’ confidentiality. Nevertheless, students had the option to provide personal information if they desired feedback on their individual results. The participants were explicitly informed that their responses would remain confidential and would not have any academic or professional consequences. These measures were intended to foster trust and encourage honest participation, given the sensitive nature of some survey items. The dissemination campaigns for the project were executed via social media platforms to maximize outreach. To ensure a structured approach for the administration of the measurement instruments, meetings were convened with the academic authorities, faculty members, and participating researchers. These meetings aimed to devise the logistics of the screening process and establish a detailed schedule specifying the dates and times for each group’s participation. Furthermore, during the student recruitment phase, project coordinators conducted in-person visits to each group to elucidate the objectives of the study, extend invitations for participation, and address queries from potential participants. All activities were undertaken with the authorization and consent of the authorities of the Academic Unit of Human Medicine and Health Sciences. Students accessed the measurement instruments via a QR code that they scanned using their mobile devices. Upon accessing the platform, participants were required to provide informed consent electronically before completing the instruments. The entire process was designed to be completed within 40 min.

### 2.5 Data quality and missing data

The online survey platform automatically routed participants to the next section if they indicated no use of substances such as tobacco, alcohol, or other drugs in the past six months, effectively serving as a built-in filter. This design minimized the risk of incomplete or invalid responses to these items. Additionally, response patterns were monitored to detect inconsistencies such as identical responses across multiple scales or short completion times. Patterned or inattentive responses were not observed. All the participants provided complete and valid responses to the inventories included in this study. The exclusion and elimination criteria specified in the protocol were not triggered because no responses were incomplete or invalid. Consequently, no participants were excluded and no missing data were observed. Because there were no missing values, the imputation methods were not applied. This ensured that the full dataset was available for analysis. To maintain anonymity, identifiable information was stored separately from survey responses using a secure coding system.

### 2.6 Ethical considerations

The protocol was reviewed and approved by the Institutional Ethical and Research committees (ID: AMMCCI-FACTOR-06, CEICANCL-12052023). This study was conducted in compliance with international ethical standards, institutional guidelines, and strict adherence to the Mexican General Health Law in the field of research, which classifies this study as without risk investigation. International Ethical Guidelines for Health-related Research Involving Humans and Helsinki Declaration. Since this study used a web-based survey, it was designed to strictly observe the Mexican Federal Law on Protection of Personal Data Held by Individuals to prevent indirect damage to the participants and to protect their data. All individuals who agreed to participate signed informed consent forms.

### 2.7 Data analysis

Parametric or non-parametric statistical tests were employed to analyze qualitative or quantitative variables, and the relevant study groups were compared. Chi-squared tests and odds ratios (ORs) were used for categorical or qualitative variables, while Student’s t-test or Mann–Whitney U test was used for quantitative variables. Data are presented as means ± standard deviation or numbers (percentages). Statistical analysis was performed using a univariate approach to identify significant predictors for inclusion in the multivariate logistic regression model. Categorical variables are transformed into dummy variables to ensure compatibility with the regression model. In the multivariate analysis, predefined hypotheses guided the selection of predictors, and the focus was placed on assessing the combined effects of these variables, rather than performing independent hypothesis tests. Multivariate logistic regression models were used to weigh the risk factors and calculate adjusted OR. An exploratory factor analysis was conducted to ascertain the interactions between the variables and reduce the dimensions. The method employed was principal component analysis based on the eigenvalues. A Varimax rotation test, the Kaiser–Meyer–Olkin (KMO) test (51), and Bartlett’s sphericity test (52) were applied as measures of sample adequacy. Statistical analyses were performed using SPSS (v.29, IBM Corporation, Chicago, IL, USA) for Windows. The components and statistics obtained from the factor analysis (SPSS) were stored in a.csv format dataset and plotted using the igraph library in the R programming language v4.3.3 (53) for visualization purposes only. Statistical significance was set at p <0.05. All statistical analyses were independently verified by two researchers to minimize errors and ensure the integrity of the results.

## 3 Results

### 3.1 General characteristics of the study population and suicide behavior stratification

The study sample consisted of 688 students from the Bachelor of General Medicine program at Autonomous University of Zacatecas. A total of 423 participants were female (61.4%) (**Table 1**). The average age was 20.5 years ( ± 2.12), with the highest percentage of students aged between 18 and 23 years. In terms of gender identity, the majority were categorized as cisgender, with 669 (97.2%) students, 11 (1.6%) as non-binary, and 4 (0.6%) as transgender. Regarding sexual orientation, most students identified as heterosexual, totaling 543 (78.9%), with 13.2% identifying as bisexual, 3.6% as homosexual, and 4.2% (pansexual, demisexual, asexual, anthrosexual, demiromantic) were classified in ‘Other’ category (**Table 1**). A significant majority of participating students (96.9%) reported being single. A total of 337 students were not from the city of Zacatecas; that is, they were non-local (foreign) and 351 were local, representing 48.9% and 51.1%, respectively. Additionally, 110 (15.9%) participants had paid jobs besides studying. Approximately 99.2% of the students reported that they did not have children. Socioeconomic status analysis revealed that 364 (52.9%) students were in the middle class, 213 (30.9%) were in the lower-middle class, 92 (13.3%) were in the upper-middle class, and 19 (2.7%) were impoverished. Evaluation of physical activity indicated that 44.7% of the participants engaged in high physical activity, 24.1% in medium physical activity, and 214 (31.1%) in low physical activity.

**Table 1:** General characteristics of the study population classified as presence and absence of suicide behavior.

Variable	Total (n = 688)	Suicide behavior		P-value
		Presence (n = 185)	Absence (n = 503)	
Age	20.5 ± 2.12	20.6 ± 2.29	20.4 ± 2.04	0.408
Sex				
Female	423 (61.4)	126 (68.1)	297 (59.0)	**0.038**
Male	265 (38.5)	59 (31.9)	206 (41.0)	
Foreigner				
Yes	337 (48.9)	85 (45.9)	252 (50.1)	0.379
No	351 (51)	100 (54.1)	251 (49.9)	
Social class				
Upper middle class	92 (13.3)	19 (10.3)	73 (14.5)	**0.0014**
Middle class	364 (52.9)	82 (44.3)	282 (56.1)	
Lower middle class	213 (30.9)	77 (41.6)	136 (27.0)	
Poverty	19 (2.7)	7 (3.8)	12 (2.4)	
Living alone				
Yes	86 (12.5)	29 (15.7)	57 (11.3)	0.162
No	602 (87.5)	156 (84.3)	446 (88.7)	
Sexual orientation				
Heterosexual	543 (78.9)	124 (67)	419 (83.3)	**<0.0001**
Bisexual	91 (13.2)	35 (18.9)	56 (11.1)	
Homosexual	25 (3.6)	6 (3.2)	19 (3.8)	
Other	29 (4.2)	20 (10.8)	9 (1.8)	
Psychiatric illness history				
Yes	232 (33.7)	93 (50.3	139 (27.6)	**<0.001**
No	456 (66.2)	92 (49.7)	364 (72.4)	
Childhood trauma history				
Yes	282 (40.9)	101 (54.6)	181 (36.0)	**<0.001**
No	174 (25.2)	25 (13.5)	149 (29.6)	
I don’t know	232 (33.7)	59 (31.9)	173 (34.4)	
Bullying				
Yes	390 (56.6)	117 (63.2)	273 (54.3)	**0.044**
No	298 (43.3)	68 (36.8)	230 (45.7)	
Marital status				
Single	667 (96.9)	182 (98.4)	485 (96.4)	0.3613
Married	7 (1)	2 (1.1)	5 (1.0)	
In a relationship	7 (1)	0 (0)	7 (1.4)	
Common-law marriage	7 (1)	1 (0.5)	6 (1.2)	
Working				
Yes	110 (15.9)	37 (20.0)	73 (14.5)	0.104
No	578 (84)	148 (80.0)	430 (85.5)	
Children				
Yes	5 (0.72)	4 (2.2)	1 (0.2)	**0.029**
No	683 (99.2)	181 (97.8)	502 (99.8)	
Physical activity				
High	308 (44.7)	73 (39.5)	235 (46.7)	0.1222
Medium	166 (24.1)	44 (23.8)	122 (24.3)	
Low	214 (31.1)	68 (36.8)	146 (29.0)	

*P <*0.05 are highlighted in bold.

To stratify suicidal behavior among the participants, the Beck scale (22), which is widely used to evaluate the severity of suicidal ideation and to determine the urgency of intervention required, was used. Considering the findings of Beck scale scores, 82.8% of the participants, fell within the ‘Normal’ category, 10.8% required further ‘Assessment,’ suggesting that their scores were indicative of a level of suicidal thoughts that warrants additional evaluation. The remaining 5.4% was categorized as needing ‘Treatment,’ highlighting a critical level of suicidal ideation/attempt that likely necessitates immediate intervention. A total of 58 (8.4%) students had a previous suicide attempt. Of these, 35 (60.3%) had one attempt and the remaining 23 (39.7%) reported that they had more than one previous suicide attempt. Considering both suicidal ideation and attempts in the same category, suicidal behavior was observed in 185 (26.9%) participants. **Table 1** presents the results of the general population comparisons between medical students with and without suicidal behaviors. Significant associations with suicidal behavior were identified for the following variables: sex, social class, sexual orientation, history of psychiatric illness, history of childhood trauma, bullying, and having children (*p <*0.05).

### 3.2 Attention-deficit/hyperactivity disorder, depression, anxiety, and stress

The results showed that 438 (63.6%) of the medical students were classified as indicative ADHD, 114 (16.5%) as probable ADHD, and 136 (19.7%) were unlikely to have ADHD. There was a strong association between ADHD symptoms and suicidal behavior in the study population (*p <*0.0001). Symptoms suggestive of ADHD increased the odds of suicide behavior 3.34-fold among the studied population (OR = 3.34; 95% CI: 2.2–5.0; *p* = 4.9 × 10^−9^).

The results of the DASS-21 tool (24) revealed that 255 (37%) students had mild depression and 181 (26.3%) had moderate depression (**Table 2**). For anxiety, the results were as follows: 193 (28%) had mild anxiety, 182 (26.4%) moderate anxiety, and 10 (1.4%) severe anxiety. For stress, the following results were reported for different severity levels: mild stress was reported in 178 (25.8%) participants, moderate stress in 165 (23.9%), and severe stress in 11 (1.5%). There was no significant association between symptoms suggestive of depression, anxiety, or stress and suicidal behavior (*p >*0.05).

**Table 2:** Results of the mental health inventories administered to the medical students classified according with presence or absence of suicide behavior.

Variable	Total (n = 688)	Suicide behavior		P-value
		Presence (n = 185)	Absence (n = 503)	
ADHD				
Unlikely	136 (19.7)	20 (10.8)	116 (23.1)	**<0.0001**
Probable	114 (16.5)	14 (7.6)	100 (19.9)	
Indicative	438 (63.6)	151 (81.6)	287 (57.1)	
Depression				
No depression	252 (36.6)	59 (31.9)	193 (38.4)	0.2749
Mild	255 (37)	72 (38.9)	183 (36.4)	
Moderate	181 (26.3)	54 (29.2)	127 (25.2)	
Anxiety				
No anxiety	303 (44)	73 (39.5)	230 (45.7)	0.4053
Mild	193 (28)	54 (29.2)	139 (27.6)	
Moderate	182 (26.4)	54 (29.2)	128 (25.4)	
Severe	10 (1.4)	4 (2.2)	6 (1.2)	
Stress				
No stress	334 (48.5)	89 (48.1)	245 (48.7)	0.9225
Mild	178 (25.8)	49 (26.5)	129 (25.6)	
Moderate	165 (23.9)	45 (24.3)	120 (23.9)	
Severe	11 (1.5)	2 (1.1)	9 (1.8)	
Impulsivity				
High impulsivity	412 (59.8)	121 (65.4)	291 (57.9)	0.088
Low impulsivity	276 (40.1)	64 (34.6)	212 (42.1)	
Hopelessness				
No hopelessness	3 (0.43)	0 (0)	3 (0.6)	**0.0001**
Mild	157 (22.8)	36 (19.5)	121 (24.1)	
Moderate	508 (73.8)	135 (73)	373 (74.2)	
Severe	20 (2.9)	14 (7.6)	6 (1.2)	
Anhedonia				
Always	83 (12.06)	44 (23.8)	39 (7.8)	**<0.0001**
Almost always	158 (22.9)	54 (29.2)	104 (20.7)	
Sometimes	336 (48.8)	71 (38.4)	265 (52.7)	
Rarely	84 (12.2)	11 (5.9)	73 (14.5)	
Never	27 (3.9)	5 (2.7)	22 (4.4)	

ADHD, Attention-deficit/hyperactivity disorder. *P <*0.05 are highlighted in bold.

### 3.3 Impulsivity, hopelessness, and anhedonia

Together with depression, impulsivity and hopelessness are factors that can lead from ideation to a suicide attempt, and they are considered part of the suicide triad. The results of the impulsivity inventory of Plutchik indicated that 412 students (59.8%) exhibited high impulsivity. The Beck Hopelessness Inventory findings showed that 20 (3%) of the students exhibited severe hopelessness, 508 (74%) moderate, and 157 (23%) mild. There were differences in the proportion of hopelessness between subjects with and without suicidal behavior (*p* = 0.0001), whereas for impulsivity, no significant differences were observed (*p* = 0.088).

When students were queried: In the last 6 months, have you felt less motivated in things that previously gave you pleasure? it was found that they exhibited varying degrees of anhedonia: in the ‘Always’ category 82 (12.06%), ‘Almost always’ 158 (22.9%), ‘Sometimes’ 336 (48.8%), ‘Rarely’ 84 (12.2%), and ‘Never’ 27 (3.9%). There were marked differences in the proportion of anhedonia between individuals with and without suicidal behavior (*p <*0.0001).

### 3.4 Self-Harm, and suicidal ideation/attempt motivations

**Figure 1:**
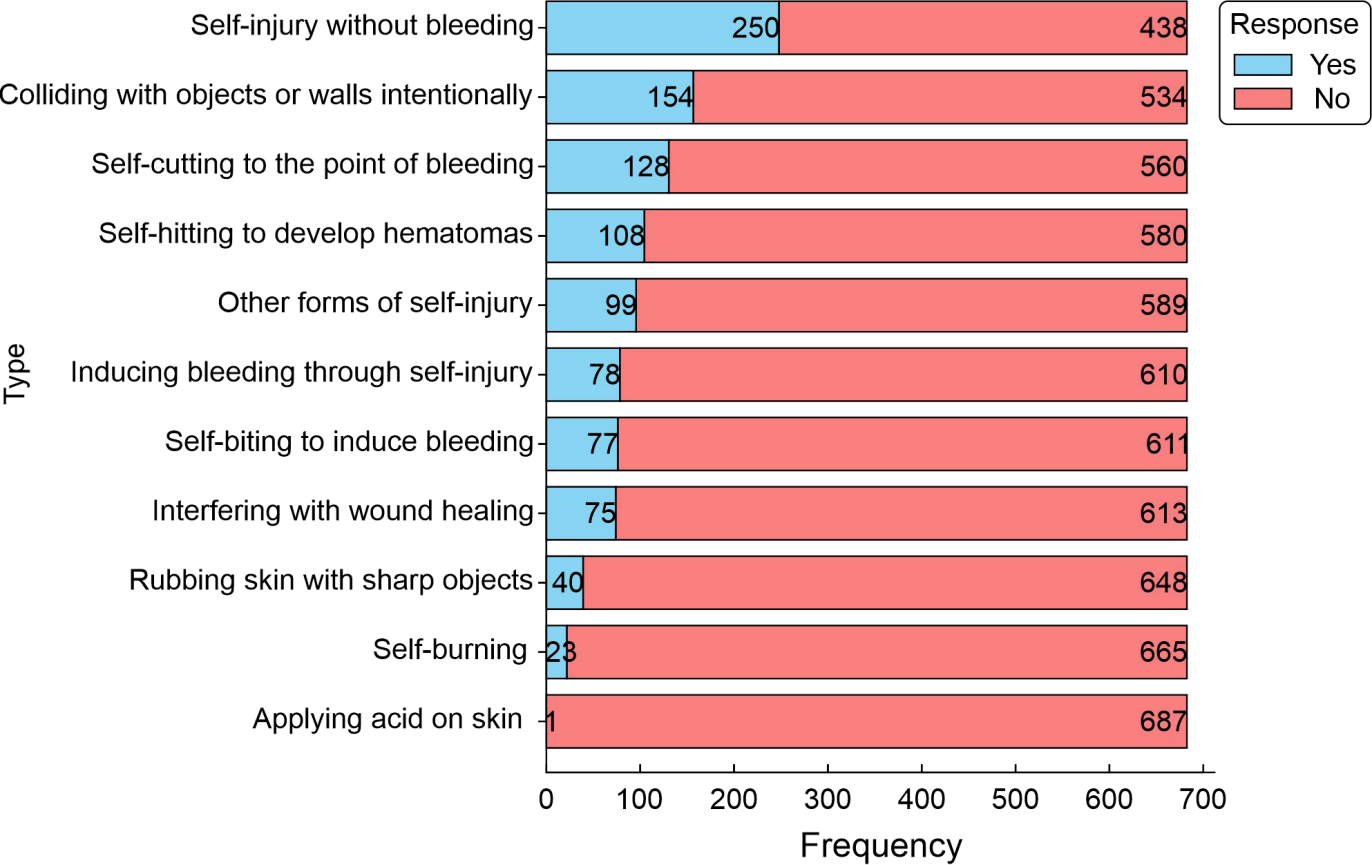
Frequency of self-harms in the study population. The graph represents the number of medical students reporting various self- harming behaviors (Yes) and the proportion of medical students who did not report these behaviors (No). The frequency indicates the number of students citing each cause (n = 688).

When assessing the frequency and severity of self-harm among the study population, it was found that none of the students exhibited severe injuries. **Figure 1** shows the types of self-hams among the participants. Approximately 36.3% of the students indicated that they scratched, marked, or pricked their skin without bleeding; 22.4% slammed their head or one of their limbs against an object or wall to hurt themselves; 18.6% cut until they bled or injured their skin; and 7.1% of the study population required treatment for self-harm. Approximately 45.8% of participants had no history of self-harm behavior.

**Figure 2:**
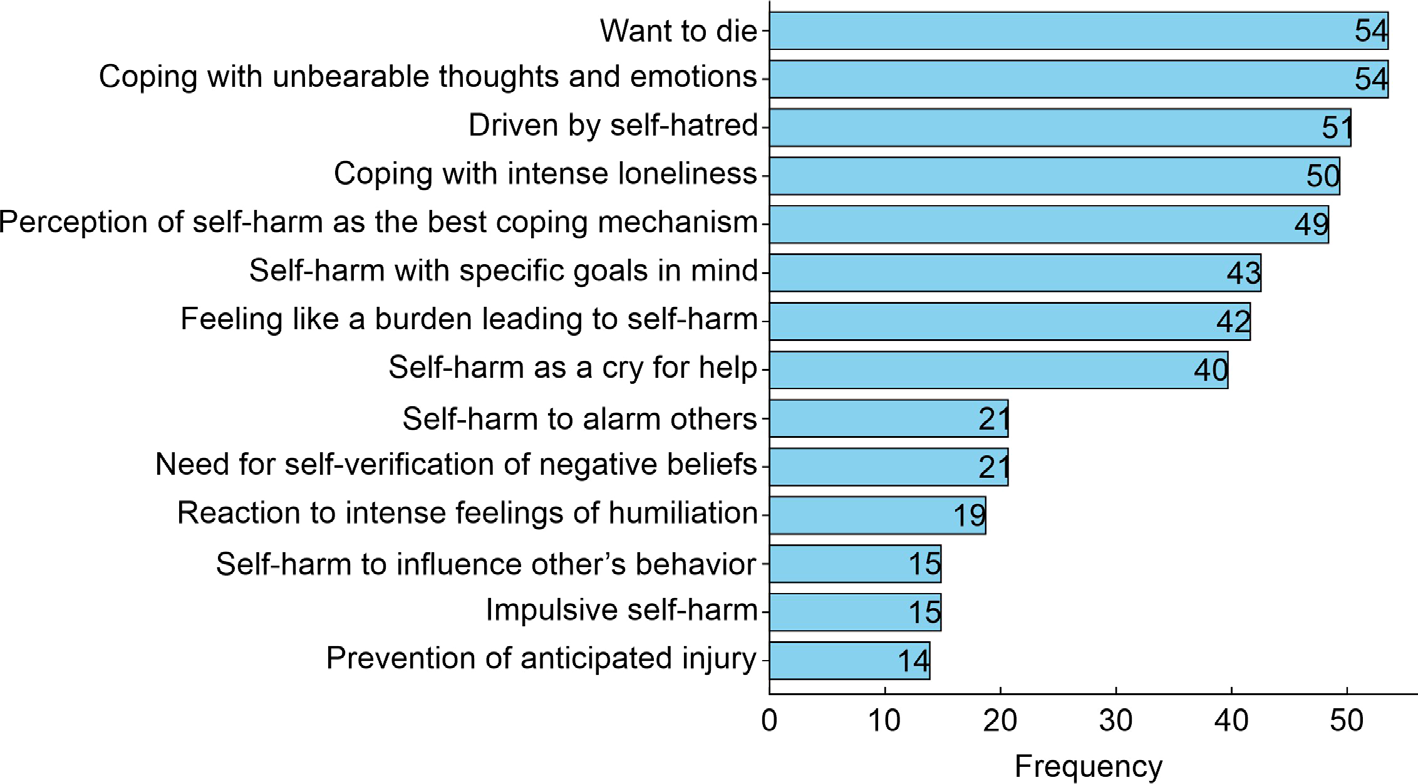
Causes and motivations leading to self-harm behavior in the study population. The graph reflects the reported motivations for self-harm among the surveyed students with suicidal behavior (n = 185). The frequency indicates the number of medical students citing each cause.

Of the 185 participants with suicidal behavior, 54 (29.2%) simply wanted to die, 54 (29.2%) manifested it as a way of coping with unbearable thoughts and emotions, 51 (27.6%) were driven by self-hatred, 50 (27%) were coping with intense loneliness, 49 (26.5%) thought self-harm was the best way to deal with their problems, 42 (22.7%) felt like a burden, and 40 (21.6%) considered self-harm a cry for help (**Figure 2**).

### 3.5 Addictions

#### 3.5.1 Tobacco and alcohol consumption

**Table 3** displays the frequencies of drug consumption, including tobacco and alcohol consumption, for all participants and the results obtained from the comparisons between the proportions of consumption between individuals with and without suicidal behavior. According to the FTND inventory (32), 44 (6.4%) students reported occasional, social, or daily tobacco consumption. No medical students scored high for a high risk of tobacco addiction, but four (0.58%) students had moderate addiction, and 40 (5.8%) had mild addiction (**Table 3**). Concerning alcohol consumption, 512 (74.4%) students reported consumption, of which 118 (23.05%) were at medium risk of addiction, 10 (1.95%) were at high risk of alcohol addiction, and four (0.78%) fell in the category of likely addiction. The proportions of both tobacco and alcohol consumption were statistically different between students with and without suicidal behavior (*p <*0.05).

**Table 3:** Comparisons of substances consumption patterns and consume situations between individuals with presence and absence of suicide behavior.

Substance	Total (n = 688)	Suicide behavior		P-value
		Presence (n = 185)	Absence (n = 503)	
Tobacco				
Without consumption	644 (93.6)	160 (86.5)	484 (96.2)	**<0.0001**
Mild	40 (5.8)	22 (11.9)	18 (3.6)	
Moderate	4 (0.58)	3 (1.6)	1 (0.2)	
Alcohol				
Low risk	556 (80.8)	134 (72.4)	422 (83.9)	**0.0059**
Medium risk	118 (17.1)	44 (23.8)	74 (14.7)	
High risk	10 (1.4)	5 (2.7)	5 (1.0)	
Likely addiction	4 (0.58)	2 (1.1)	2 (0.4)	
Cannabis				
Without symptoms of addiction	653 (94.9)	168 (90.8)	485 (96.4)	**0.0102**
With symptoms of problematic use	19 (2.7)	10 (5.4)	9 (1.8)	
Symptoms of addiction	16 (2.3)	7 (3.8)	9 (1.8)	
Consume situations	333 (40.4)	98 (52.97)	235 (46.2)	NA
Pleasant emotions*				
>50%	47 (14.1)	17 (17.35)	30 (12.8)	0.3568
<0%	286 (85.9)	81 (82.65)	205 (87.2)	
Self-control*				
>50%	4 (1.2)	1 (1)	3 (1.3)	0.7216
<50%	329 (98.8)	97 (99)	232 (98.7)	
Physical discomfort*				
>50%	4 (1.2)	3 (3.1)	1 (0.4)	0.1443
<50%	329 (98.8)	95 (96.9)	234 (99.6)
Need or craving*				
>50%	2 (0.6)	2 (2)	0 (0)	0.1561
<50%	331 (99.4)	96 (98)	235 (100)	
Unpleasant emotions*				
>50%	21 (6.3)	14 (14.3)	7 (3)	**0.0003**
<50%	312 (93.7)	84 (85.7)	228 (97)	
Pleasant moments with others*				
>50%	52 (15.6)	19 (19.4)	33 (14)	0.2896
<50%	281 (84.4)	79 (80.6)	202 (86)	
Conflict with others*				
>50%	6 (1.8)	6 (6.1)	0 (0)	**0.0007**
<50%	327 (98.2)	92 (93.9)	235 (100)	
Social pressure*				
>50%	4 (1.20)	1 (1)	3 (1.3)	0.7216
<50%	329 (98.8)	97 (99)	232 (98.7)	

*Percentages of each category were calculated considering only the students with history of substance consumption (n total = 333; presence of suicidal behavior: n = 98; absence of suicidal behavior: n = 235). NA, Not applicable. *P <*0.05 are highlighted in bold.

#### 3.5.2 Cannabis, cocaine, and other drugs

Of all the participants, 123 (17.87%) reported using cannabis. Approximately 16 (2.3%) individuals had symptoms of addiction to this substance and 19 (2.8%) exhibited problematic use. The frequency of cannabis consumption in the categories of without addiction, with symptoms of problematic use, and with symptoms of addiction were 24.4%, 1.5%, and 1%, respectively, in the group exhibiting suicidal behavior and 70.5%, 1.3%, and 1.3%, respectively, in the group without suicidal behavior (*p <*0.05). In the study population, only five (0.73%) students reported a history of cocaine use, and none showed any degree of addiction to this substance.

**Figure 3:**
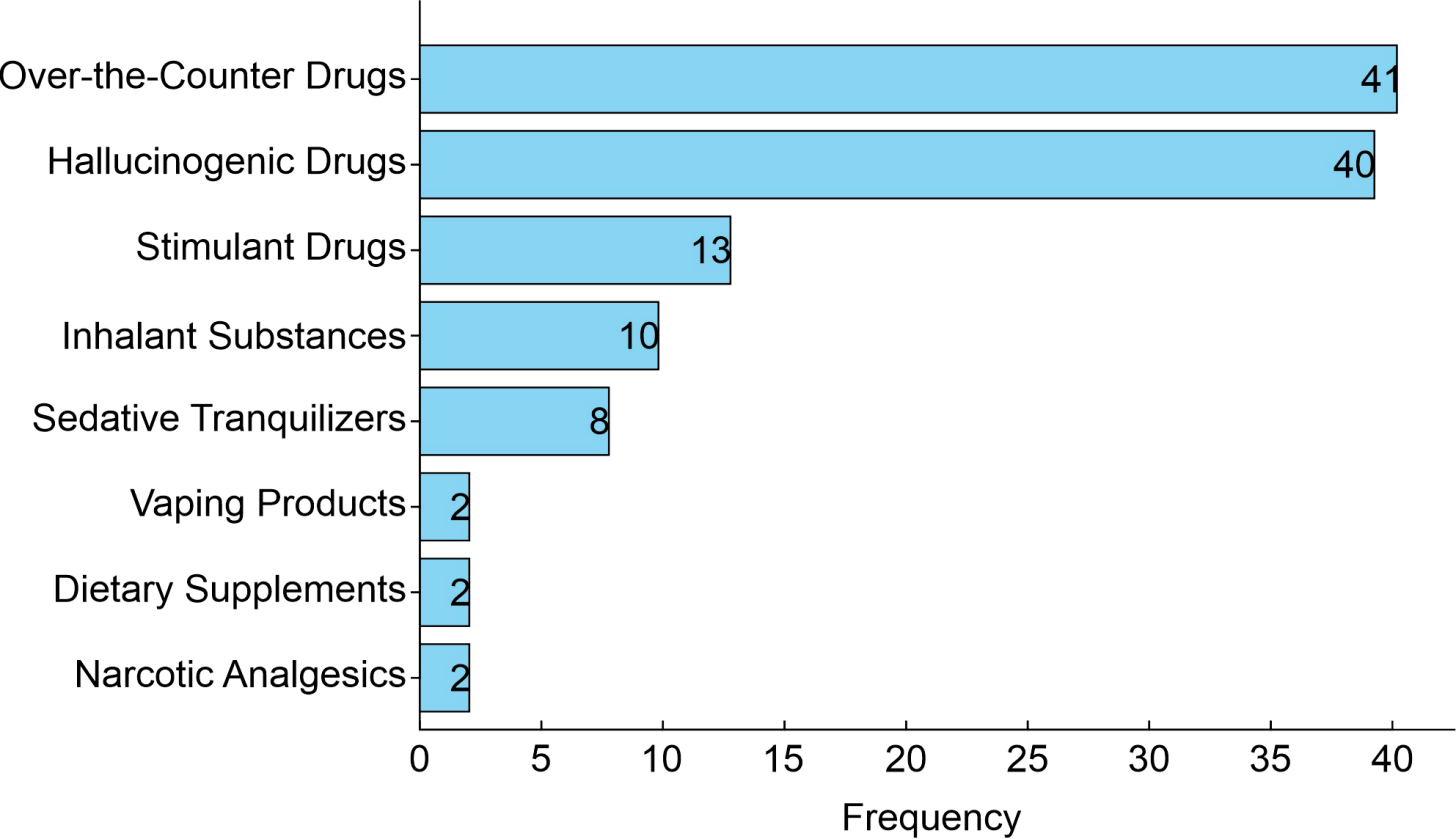
Frequency of other drugs used by medical students. Data represent the frequency of reported usage of each drug category among the participants in the study (n = 118).

In addition to the aforementioned drugs, the use or current consumption of a wider range of drugs has also been investigated. **Figure 3** shows the frequency of the most common drugs used by students. A total of 40 (5.8%) students were found to consume hallucinogens [LSD (acid), mescaline, peyote, mushrooms, ecstasy, MDA, MDMA, bufotenin, ayahuasca, psilocybin, PCP (angel dust, peace pill), STP], and 41 (5.9%) over-the-counter medications [steroids, methylphenidate, modafinil, diet, or sleeping pills (benzodiazepines), and carbamazepine].

#### 3.5.3 Situations of substance consumption

**Table 3** displays the circumstances or instances in which the students used both legal and illegal substances. The primary cause of consumption was pleasant moments with others (15.6%), followed by pleasant (14.1%) and unpleasant emotions (6.3%). Substance consumption patterns linked to unpleasant emotions (*p* = 0.0003) and conflict with others were associated with suicidal behavior (*p* = 0.0007).

### 3.6 Perceived social support

Regarding social support, the following results were obtained during univariate analysis (**Table 4**): for material assistance, two (0.29%) students perceived minimal support, 161 (23.3%) medium, and 525 (76.3%) perceived maximum support. In the emotional/informational support section, 221 (32.1%) students were recorded as having medium support, and five (0.72%) as minimal. In the affective support section related to expressions of love and affection, students who perceived maximum support were 567 (82.4%), medium 117 (17%), and minimal four (0.58%) individuals. The perception of minimal support for social relationships of leisure and distraction was expressed by two (0.29%), medium support by 128 (18.6%), and 558 (81.1%) perceived maximum support. For all dimensions of this inventory, there were significant differences in the proportions of individuals with maximum support and those observed in minimum/medium support between groups with and without suicidal behavior (OR = 1.97–3.1; *p <*0.001).

**Table 4:** Social support findings and their comparison between medical students with and without suicidal behavior.

Variable	Total (n = 688)	Suicide behavior		P-value*	OR	95% CI
		Presence (n = 185)	Absence (n = 503)			
Global social support index	17.6 ± 7.3	16.0 ± 7.10	18.3 ± 7.3	**<0.0001**	NA	NA
Material assistance						
Maximum	525 (76.3)	115 (62.2)	410 (81.5)	**<0.0001**	2.68	1.84–3.9
Medium	161 (23.3)	68 (36.8)	93 (18.5)			
Minimum	2 (0.29)	2 (1.1)	0 (0)			
Emotional/Informational support						
Maximum	462 (67.1)	100 (54.1)	362 (72.0)	**<0.0001**	2.1	1.45–2.92
Medium	221 (32.1)	81 (43.8)	140 (27.8)			
Minimum	5 (0.72)	4 (2.2)	1 (0.2)			
Affective support						
Maximum	567 (82.4)	128 (69.2)	439 (87.3)	**<0.0001**	3.1	2.03–4.59
Medium	117 (17)	55 (29.7)	62 (12.3)			
Minimum	4 (0.58)	2 (1.1)	2 (0.4)			
Social relations of leisure and distraction						
Maximum	558 (81.1)	136 (73.5)	422 (83.9)	**0.0013**	1.97	1.31–2.94
Medium	128 (18.6)	47 (25.4)	81 (16.1)			
Minimum	2 (0.29)	2 (1.1)	0 (0)			

*P-value refers to OR which was calculated by the comparison of ‘Maximum’ category vs. the sum of categories ‘Minimum’ and ‘Medium.’ NA, Not applicable. *P <*0.05 are highlighted in bold.

### 3.7 Multivariate data modeling to weigh variables associated with suicidal behavior and suicide attempt

To identify and weigh the risk factors associated with suicidal behavior, a multivariate logistic regression analysis was performed. In this analysis, the presence of suicidal behavior, described as the presence of ideation and/or intent, was considered as the dependent variable. All variables with *p <*0.05 in the univariate analysis were considered during the analysis. **Table 5** displays the findings and their relationships with suicidal behavior in the study population.

**Table 5:** Multivariate logistic regression analysis of variables associated with suicidal behavior.

Variable	Category	Coeff	Standard Error	Wald Statistic	P value	Odds Ratio	95% CI
	Constant	−2.41	0.285	71.3	<0.001	0.09	0.1–0.2
Tobacco use	None (Reference)						
	Mild	1.236	0.368	11.3	**<0.001**	3.44	1.67–7.08
	Moderate	1.876	1.177	2.5	0.111	6.53	0.65–65.5
Alcohol consumption	None (Reference)						
	Mild risk	0.744	0.245	9.3	**0.002**	2.11	1.30–3.4
	High risk	0.761	0.763	0.99	0.319	2.14	0.48–9.55
	Probable addiction	0.571	1.183	0.2	0.629	1.77	0.17–18.0
Sex	Female	0.445	0.212	4.4	**0.036**	1.56	1.03–2.36
Sexual orientation	Heterosexual (Reference)						
	Homosexual	0.714	0.438	2.7	0.103	2.04	0.87–4.82
	Bisexual	0.483	0.269	3.2	0.072	1.62	0.96–2.75
	Other*	2.172	0.494	19.4	**<0.001**	8.78	3.34–23.1
Family history of mental diseases	No (Reference)						
	Yes, without diagnosis	0.257	0.285	0.81	0.368	1.29	0.74–2.26
	Yes, with diagnosis	0.451	0.214	4.4	**0.035**	1.57	1.03–2.39
Hopelessness	No/Mild (Reference)						
	Moderate	0.115	0.232	0.2	0.622	1.12	0.71–1.77
	Severe	2.085	0.559	13.9	**<0.001**	8.04	2.69–24.0
Material support	Maximum (Reference)						
	Minimum/medium	0.576	0.24	5.8	**0.016**	1.78	1.11–2.85
Affective support	Maximum (Reference)						
	Minimum/medium	0.877	0.263	11.1	**<0.001**	2.40	1.44–4.00

During the analysis, the presence of suicidal behavior, described as the presence of ideation and/or intent, was considered as dependent variable. *Pansexual, demisexual, asexual, anthrosexual, and demiromantic. Coeff, Coefficient. P <0.05 are highlighted in bold.

Compared to those who did not consume tobacco, mild consumption was significantly associated with a 3-fold increase in the odds of suicidal behavior (OR = 3.442; 95% CI: 1.7–7.1, *p <*0.001), while moderate consumption, despite a higher coefficient (1.9), was not statistically significant (*p* = 0.11). Alcohol consumption at a ‘Mild risk level’ increased the odds of suicidal behavior in the study population by two times (OR = 2.105; 95% CI: 1.3–3.4, *p* = 0.002). For the sex variable, being female increased the odds of suicidal behavior 1.56 times among the study population (*p* = 0.036). The sexual orientation category of ‘Other,’ which grouped sexual orientations such as pansexual, demisexual, asexual, anthrosexual, and demiromantic, showed a strong effect on suicidal behavior with a 9-fold increase in odds (OR = 8.98; 95% CI: 3.42–23.60, *p <*0.001). Material and affective support at the minimum and medium levels were associated with increased odds of suicidal behavior (*p <*0.05), with ‘Minimum’ affective support showing a particularly strong effect (OR = 2.4, *p <*0.001). Severe hopelessness was associated with a significant increment in the odds of the outcome by 8.04 times (OR = 8.043; 95% CI: 2.7–24.0, *p <*0.001). Family history of mental diseases was also associated with increased odds of suicidal behavior (OR = 1.57; 95% CI: 1.03–2.39, *p* = 0.035).

To identify predictors related to suicide attempts, the participants without suicidal behavior and those with suicidal ideation were grouped in the same category, and suicide attempts were considered a dependent variable in statistical approximation. **Table 6** presents the results of the model. Cannabis consumption (problematic), sexual orientation (bisexual and other), anhedonia (always), family history of mental health diseases (with or without diagnosis), material support (minimum/medium), and affective support (minimum/medium) significantly increased the odds of suicide attempts in the range of 1.962 to 9.921 (*p <*0.05). Being anhedonia (‘Always’ category), sexual orientation (‘Other’ category), and problematic consumption of cannabis, the findings with the higher OR values: 9.92, 6.49, and 5.56, respectively (**Table 6**).

**Table 6:** Multivariate logistic regression analysis of variables associated with suicide attempt.

Variable	Category	Coeff	Standard Error	Wald Statistic	P value	Odds Ratio	95% CI
	Constant	−3.99	1.059	14.159	<0.001	0.02	0.0–0.1
Cannabis consumption	None (Reference)						
	Symptoms of problematic use	1.72	0.547	9.826	**0.002**	5.56	1.9–16.3
	Symptoms of addiction	1.01	0.611	2.748	0.097	2.75	0.8–9.1
Sexual orientation	Heterosexual (Reference)						
	Homosexual	0.82	0.459	3.208	0.073	2.28	0.9–5.6
	Bisexual	1.02	0.285	12.724	**<0.001**	2.76	1.6–4.8
	Other*	1.87	0.516	13.123	**<0.001**	6.49	2.4–17.9
Anhedonia	Never (Reference)						
	Rarely	−0.09	1.214	0.0054	0.942	0.92	0.1–9.9
	Sometimes	0.98	1.071	0.832	0.362	2.66	0.3–21.7
	Almost always	1.58	1.074	2.174	0.14	4.87	0.6–40
	Always	2.3	1.087	4.454	**0.035**	9.92	1.2–83.6
Family history of mental diseases	No (Reference)						
	Yes, without diagnosis	0.84	0.311	7.301	**0.007**	2.32	1.3–4.3
	Yes, with diagnosis	0.67	0.261	6.66	**0.01**	1.96	1.2–3.3
Material support	Maximum (Reference)						
	Minimum/medium	0.77	0.321	5.709	**0.017**	2.15	1.2–3.7
Affective support	Maximum (Reference)						
	Minimum/medium	1.26	0.393	10.349	**0.001**	3.54	0.9–2.9

In the statistical approximation the participants without suicide behavior and that with suicidal ideation were grouped in the same category and the suicide attempt, was considered as dependent variable. *Pansexual, demisexual, asexual, anthrosexual, and demiromantic. Coeff, Coefficient. *P <*0.05 are highlighted in bold.

### 3.8 Exploratory factor analysis for interdependence between variables

Multivariate factor analysis was conducted on the complete set of variables to reduce unobservable latent dimensions (factors) that could identify the relationships established between the variables. The results are presented in **Table 7**. The best fit (KMO = 0.608, *p <*0.01, df = 66), was observed for the following set of variables: substance use situations, physical activity, tobacco consumption, alcohol consumption, sexual orientation, socioeconomic level, living alone, foreigner, stress, depression, anxiety, and ADHD.

**Table 7:** Principal component analysis.

Component	Initial auto values		
	Total	Variance (%)	Accumulated variance (%)
1	2.722	22.684	22.684
2	1.964	16.370	39.053
3	1.472	12.264	51.318
4	1.288	10.731	62.049
5	1.075	8.960	**71.009**
6	0.845	7.038	78.047
7	0.733	6.105	84.151
8	0.638	5.314	89.465
9	0.487	4.057	93.522
10	0.354	2.946	96.468
11	0.323	2.694	99.162
12	0.101	.838	100.000

Total explained variance is displayed. Cases where suicidal behavior was present were used in this analysis stage. Value in bold indicate the accumulated variance for the components with initial auto values higher than 1 in data the modeling.

The communalities, which estimate the variance in each variable accounted for by the factors in the factor solution, are displayed in **Supplementary Table S1**, and the explained variance ratios (the percentage of variance that is attributed to each of the selected components) are shown in **Table 7**.

The principal component analysis suggested five components (accumulated variance of 71.01%). The component matrix (**Supplementary Table S2**) and the rotated component matrix (**Table 8**) show the loadings for each variable on each component. This factor analysis grouping was verified using a varimax orthogonal rotation. The rotated component matrix helps to determine what the components represent. It contains Pearson correlations between items and components. The components were: Component 1: Stress, Depression, Anxiety, and ADHD. Component 2: Physical Activity, and Tobacco. Component 3: Living alone and Foreigner. Component 4: Substance use situations and alcohol consumption. Component 5: Sexual orientation and socioeconomic status.

**Table 8:** Factor analysis rotated component matrix (varimax).

Variable	Component				
	1	2	3	4	5
Stress	**.837**	–	–	–	–
Depression	**.836**	–	–	.126	.107
Anxiety	**.819**	–	.110	.121	
ADHD	**.658**	–	−.115	–	−.115
Tobacco consumption	–	**.964**	–	–	–
Physical activity	–	**.961**	–	–	–
Foreigner	–	–	**.844**	−.134	–
Living alone	–	–	**.816**	.127	–
Substance use situations	–	–	–	**.822**	
Alcohol consumption	–	−.126	–	**.784**	.123
Sexual orientation	.139	–	−.183	–	**.767**
Socioeconomic level	−.205	−.148	.238	–	**.713**

During the construction of the matrix, small values were omitted. Only cases where suicidal behavior value equals 1 (presence) were used in this analysis stage. Values with more weight for each component are highlighted in bold.

**Figure 4:**
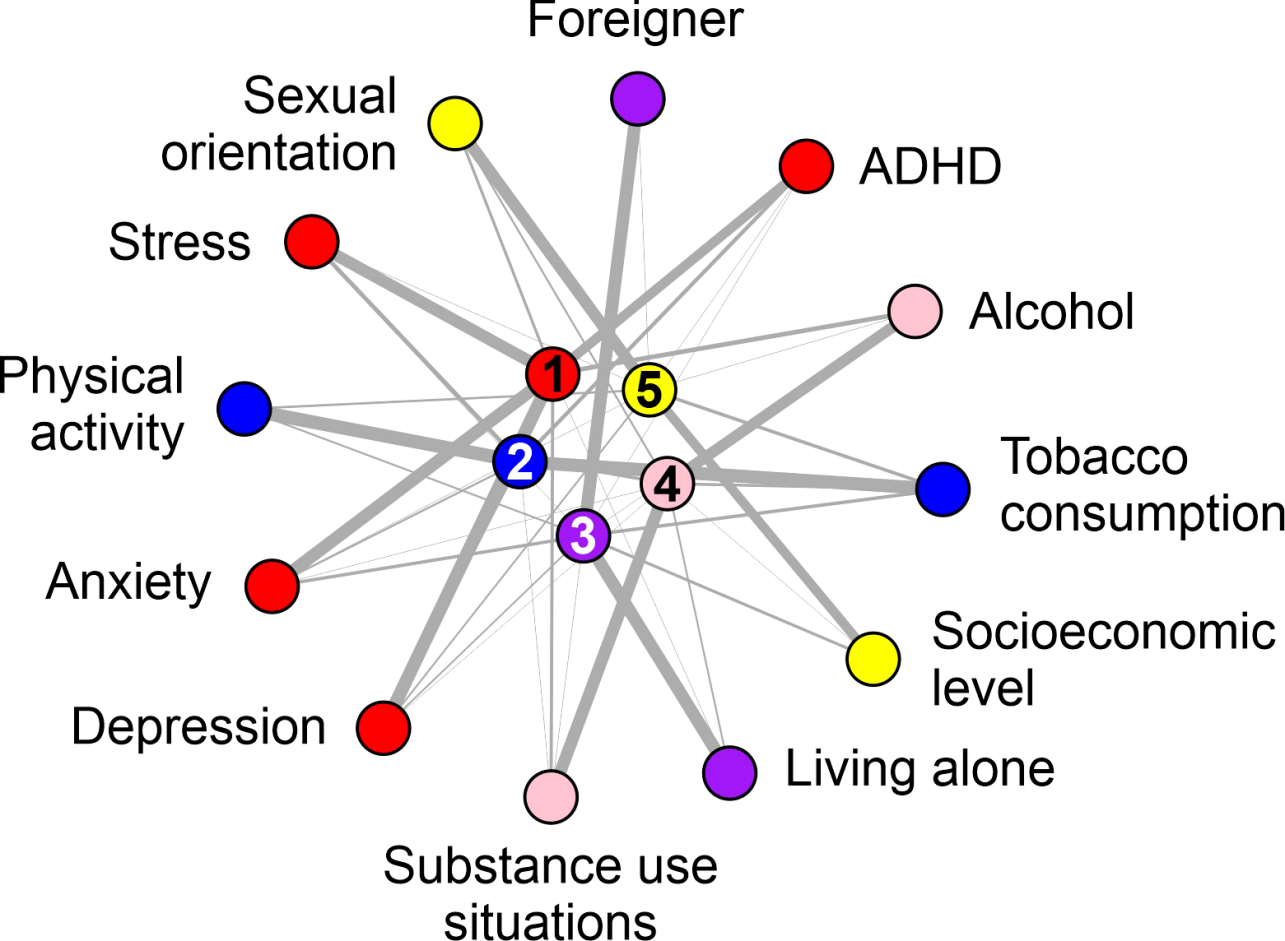
Graphical representation of the principal component analysis. The data visualization from the factor analysis (principal components) was constructed using the igraph library in the R programming language. Each component is indicated by a number (1 to 5) and one color (red, blue purple, red, and yellow). The variables with the highest weights inside each component are colored in the same color.

**Figure 4** graphically represents the relationship between the variables and components derived from the principal component analysis. Each variable and component are depicted as nodes, while the correlations between them are illustrated by vertices. The width of each vertex indicates the strength of the correlation; a thicker line signifies a strong correlation between a variable and a component, whereas a finer line indicates a weaker correlation. In this visual representation, different colors are used to distinguish the components, with each color corresponding to a specific component number (1–5). The variables with the highest weights within each component are highlighted in the same color, providing a clear visual indication of their significance. This graphical approach simplifies the understanding of complex relationships and emphasizes the most influential factors within each component.

## 4 Discussion

Suicidal behavior has a significant impact not only on the individual but also on their familiar surroundings, academic communities, and society as a whole. The identification of risk factors associated with suicidal behavior in medical students remains a matter of great importance because of the increased stress and mental health challenges faced by this population (8, 12). This study provides new insights into the multifaceted nature of suicidal behavior, highlighting the interplay between individual characteristics, mental health conditions, substance use, and social support.

Our results revealed that women had a higher association with suicidal behavior but not with suicide attempts, suggesting potential sex-specific risk factors. Sexual orientation was a significant factor, with individuals categorized as ‘Other’ showing an odds of 8.78 suicidal behavior, indicating they were nearly nine times more likely to engage in such suicidal behavior. Bisexuality was associated with suicide attempts, and, while not reaching statistical significance, homosexual individuals also exhibited increased odds. These findings are consistent with prior research indicating that sexual minorities face elevated levels of discrimination and mental health challenges, increasing their vulnerability to suicidal behavior (54).

Mental health plays a central role. A surprisingly high percentage of participants (63%) were classified as having indicative ADHD, which was significantly associated with suicidal behavior (*p <*0.0001). It is important to note that “indicative ADHD” is derived directly from the ASRS classification system, which was used in this study as a screening tool and does not have diagnostic value. In students, ADHD symptoms, particularly impulsivity and inattention, may contribute to chronic frustration, emotional dysregulation, and difficulties in coping with stress, all of which are well-documented risk factors for hopelessness and suicidal ideation (6, 55). Additionally, anhedonia further compounds this risk by depriving individuals of natural rewards that encourage positive engagement with life. The potential overlap of ADHD symptoms with those of anxiety and depression could also explain the higher prevalence observed in our sample than in other local (27.9%) (16) or global estimates (2.58%–6.76%) (56). This discrepancy may reflect the unique stressors experienced by medical students, which can exacerbate the symptoms of inattention and hyperactivity. Medical schools in Mexico should prioritize screening mechanisms for this disorder to improve the academic trajectories of these students and their quality of life and to prevent future suicidal behaviors and the consequent negative impacts on their academic communities.

Substance use was also a significant contributor. Mild tobacco use (OR = 3.44, *p <*0.001) and mild alcohol consumption (OR = 2.11, *p* = 0.002) were associated with increased odds of suicidal behavior. Contextual factors surrounding substance use also played a role: consumption in response to unpleasant emotions (*p* = 0.0003) and interpersonal conflicts (*p* = 0.0007) highlighted the importance of addressing the circumstances in which substances are used. The association between smoking and suicidal behavior is consistent with previous research suggesting that nicotine withdrawal exacerbates the underlying psychiatric conditions (57, 58). These findings indicate the need for interventions that not only target substance use, but also address the emotional triggers behind it.

In our study, all dimensions of perceived social support (material assistance, emotional/informational support, affective support, and social relations) showed differences between students with and without suicidal behavior. In particular, material and affective support were associated with a reduced likelihood of suicidal behavior in the multivariate analysis. This aligns with previous studies demonstrating the protective role of strong social support networks in preventing mental health issues (59, 60). Although included in the analysis, emotional support did not remain significant in the multivariate model, suggesting an indirect or less prominent role in the context examined.

Interestingly, cannabis use, sexual orientation (bisexual and other), and anhedonia emerged as significant predictors of suicide attempts in the multivariate models, whereas tobacco and alcohol consumption, as well as sex, remained significant. These results emphasize the complexity of the transition from suicidal ideation to attempts and highlight the need for interventions that address specific risk factors in this progression.

The factor analysis conducted in this study grouped related variables into components, such as depression, anxiety, stress, and ADHD (component 1), and situations of substance use and alcohol consumption (component 4), which align with patterns identified in the literature (6, 61, 62). These components underscore the potential explanatory strength of these clusters in predicting suicidal behavior and provide a framework for developing new tools to assess suicide risk in medical students. However, the cross-sectional design limits our ability to establish causality or disentangle contributions, such as ADHD symptoms versus comorbid conditions, such as anxiety or depression. Future longitudinal studies are needed to confirm these findings and to explore the dynamic interplay of these variables over time.

The multifaceted nature of suicidal behavior, including ADHD symptoms, hopelessness, anhedonia, and factors such as gender, substance use, and sexual orientation, calls for integrated interventions that simultaneously address mental health, substance use, and enhancement of social support systems. Educational institutions, particularly medical schools, have an ethical responsibility to implement routine screenings for mental health symptoms and substance use, thus providing early support for high-stress populations. Public health policies in Mexico should prioritize reducing stigma surrounding mental health care and substance use treatment, fostering academic environments that promote well-being. By addressing these overlapping risk factors and strengthening social support systems, institutions can not only mitigate the risk of suicidal behavior but also ensure the development of competent, ethical, and mentally healthy professionals.

### 4.1 Limitations and future directions

Some study limitations should be highlighted: 1. The cross-sectional design restricts causal inferences between variables and suicidal behavior, and only identifies associations. Temporal variability in the conditions of students cannot be captured, necessitating longitudinal research for a more comprehensive understanding. 2. The sample of medical students limits the generalizability to other populations, and self-administered questionnaires may also introduce potential self-report bias. 3. The evaluation of childhood trauma and other past events relies on participant recall, which may be subject to memory biases and affect data accuracy. 4. Similarly, the absence of a “prefer not to answer” option may have influenced participants’ willingness to respond honestly, potentially introducing social desirability or self-preservation biases. While filter questions minimize unnecessary responses, future research should consider including explicit opt-out options and reinforcing participants’ education about confidentiality to reduce potential biases and enhance response validity. 5. Although this study did not include specific cultural variables, such as familism, machismo/marianismo, collectivism, religiosity, socioeconomic status, migration, and stigma, their potential impact on mental health outcomes among Mexican young adults cannot be overlooked. The complexity of evaluating these constructs across a diverse student population, along with the limitations of available standardized measures, underscores the need for future research to explore these aspects in greater depth. Investigating these variables could provide important insights into the cultural dimensions influencing suicidal behavior and resilience, ultimately contributing to more culturally sensitive mental health interventions.

## Data Availability

The original contributions presented in the study are included in the article/**Supplementary Material**, further inquiries can be directed to the corresponding author/s.
