# Supplementary material for: The hidden risk factors behind of suicidal behavior in medical students: a cross-sectional cohort study in Mexico
Source: Front Psychiatry. 2025 Mar 5;16:1505088. doi: 10.3389/fpsyt.2025.1505088 (PMC11920192; doi:10.3389/fpsyt.2025.1505088)
Supplement: Supplementary file 1 [file Table1.docx]

**Supplementary file**

**Table S1.** Communalities. Extraction method used: Principal component analysis. Cases where suicidal behavior was present were used in this analysis stage.

|  | Inicial | Extraction |
| --- | --- | --- |
| Substance use situations | 1.000 | .683 |
| Physical activity | 1.000 | .942 |
| Tobacco consumption | 1.000 | .938 |
| Alcohol consumption | 1.000 | .654 |
| Sexual orientation | 1.000 | .646 |
| Socioeconomic level | 1.000 | .629 |
| Living alone | 1.000 | .689 |
| Foreigner | 1.000 | .740 |
| Stress | 1.000 | .707 |
| Depression | 1.000 | .731 |
| Anxiety | 1.000 | .703 |
| ADHD | 1.000 | .460 |

**Table S2.** Component Matrix. Small values were omitted.

| Variable | Component | | | | |
| --- | --- | --- | --- | --- | --- |
|  | 1 | 2 | 3 | 4 | 5 |
| Depression | **.826** | .142 | .115 | - | .120 |
| Anxiety | **.798** | .135 | .215 | - | - |
| Stress | **.763** | .280 | - | -.192 | - |
| ADHD | **.616** | .252 | - | -.116 | - |
| Physical activity | -.358 | **.855** | .127 | .212 | .150 |
| Tobacco consumption | -.333 | **.834** | .221 | .184 | .221 |
| Foreigner | -.119 | -.249 | **.802** | -.142 | - |
| Living alone | - | -.116 | **.801** | .122 | -.126 |
| Substance use situations | .205 | - | - | **.772** | -.200 |
| Alcohol | .297 | -.241 | - | **.711** | - |
| Sexual orientation | .185 | -.139 | -.130 | .131 | **.747** |
| Socioeconomic level | -.142 | -.448 | .194 | - | **.606** |

Only cases where Suicidal behavior was present were used in the analysis phase.
